# Efforts to increase the racial and ethnic and socioeconomic diversity of the Adult Changes in Thought study cohort

**DOI:** 10.1002/alz.088433

**Published:** 2025-01-09

**Authors:** Nicole M Gatto, Andrea Z. LaCroix, Sarah E. Tom, James Ralston, Paul K. Crane, Roxanne Muiruri, Sundary Sankaran, Pamela A. Shaw, Laura E. Gibbons, Linda K. McEvoy

**Affiliations:** ^1^ Kaiser Permanente Washington, Seattle, WA USA; ^2^ Herbert Wertheim School of Public Health and Human Longevity Science, University of California, San Diego, La Jolla, CA USA; ^3^ University of California San Diego, La Jolla, CA USA; ^4^ Columbia University Irving Medical Center, New York, NY USA; ^5^ Kaiser Permanente Washington Research Institute, Seattle, WA USA; ^6^ University of Washington, Seattle, WA USA; ^7^ Kaiser Permanente Washington Health Research Institute, Seattle, WA USA; ^8^ University of Washington, School of Medicine, Seattle, WA USA; ^9^ University of Washington Alzheimer’s Disease Research Center, University of Washington School of Medicine, Seattle, WA USA

## Abstract

**Background:**

The Adult Changes in Thought (ACT) study is an ongoing population‐based prospective cohort study that began in 1994 and aims to identify risk and preventive factors for dementia. ACT randomly selects and enrolls Kaiser Permanente Washington (KPWA) health plan members age ≥ 65 years. Historically, the cohort make up has been 88% non‐Hispanic White participants. A core aim of the ACT U19 is to expand enrollment with a specific emphasis on increasing racial, ethnic, and socioeconomic diversity of study participants. A set target is to enroll 600 new members from historically minoritized groups by April 2026, including Asian, Black and Hispanic participants, such that these groups represent > 20% of the active sample (n = 3,000).

**Method:**

To meet our target, we implemented a series of changes to our recruitment processes including revising recruitment materials to improve their informativeness, oversampling racial and ethnic groups, and locating a second ACT research clinic site in the socioeconomically diverse southeastern region of Seattle to expand our catchment area. We will examine how our newly enrolled cohort members compare to the KPWA population with respect to age, sex, area deprivation index (ADI) and health status.

**Result:**

For the one year‐period spanning December 2022 through December 2023 when modifications to recruitment began to be rolled out and participant visits were restarted after COVID‐19, we enrolled 320 new participants with a racial and ethnic distribution of 25% Asian, 12% Black, 16% Hispanic, 2% Pacific Islander or Native American and 49% White.

**Conclusion:**

Broader representation of diverse participants within ACT will make study findings more generalizable to the KPWA source population.

